# Systematic Review: Overlap Between Eating, Autism Spectrum, and Attention-Deficit/Hyperactivity Disorder

**DOI:** 10.3389/fpsyt.2019.00708

**Published:** 2019-10-10

**Authors:** Kathrin Nickel, Simon Maier, Dominique Endres, Andreas Joos, Viktoria Maier, Ludger Tebartz van Elst, Almut Zeeck

**Affiliations:** ^1^Section for Experimental Neuropsychiatry, Department of Psychiatry and Psychotherapy, Medical Center—University of Freiburg, Faculty of Medicine, University of Freiburg, Freiburg, Germany; ^2^Department of Psychosomatic Medicine and Psychotherapy, Medical Center—University of Freiburg, Faculty of Medicine, University of Freiburg, Freiburg, Germany; ^3^Department of Psychotherapeutic Neurology, Kliniken Schmieder, Gailingen, Germany

**Keywords:** anorexia nervosa, bulimia nervosa, binge eating disorder, autism spectrum disorder, attention-deficit/hyperactivity disorder

## Abstract

**Background:** Links between eating disorders (EDs) [e.g., anorexia nervosa (AN), bulimia nervosa (BN), and binge eating disorder (BED)] and the major neurodevelopmental disorders of autism spectrum disorder (ASD) and attention-deficit/hyperactivity disorder (ADHD) have been repeatedly highlighted. In both ASD and ADHD, these links range from an elevated risk for EDs to common symptomatic overlaps and etiological commonalities with EDs.

**Methods:** We performed a systematic literature search (through July 2019) with Medline via Ovid for epidemiological data on EDs (AN, BN, and BED) in combination with both ASD and ADHD.

**Results:** The reviewed studies showed that, on average, 4.7% of patients with certain ED diagnoses (AN, BN, or BED) received an ASD diagnosis. Reliable data on the prevalence of EDs in ASD samples are still scarce. Comorbid ASD is most commonly diagnosed in patients with AN. The prevalence of ADHD in EDs ranged between 1.6% and 18%. Comorbid ADHD was more often reported in the AN-binge eating/purging subtype and BN than in the AN restrictive subtype. The prevalence of EDs in ADHD ranged between no association and a lifetime prevalence of 21.8% of developing an ED in women with ADHD.

**Conclusions:** Studies on the prevalence rates of EDs in ADHD and ASD and *vice versa* are heterogeneous, but they indicate frequent association. While there is growing evidence of clinical overlaps between the three disorders, it remains difficult to determine whether overlapping characteristics (e.g., social withdrawal) are due to common comorbidities (e.g., depression) or are instead primarily associated with EDs and neurodevelopmental disorders. Furthermore, prospective studies are required to better understand how these disorders are related and whether ADHD and ASD could be either specific or nonspecific predisposing factors for the development of EDs.

## Introduction

The exact relationship and overlap between eating disorders (EDs) [e.g., anorexia nervosa (AN), bulimia nervosa (BN), and binge eating disorder (BED)] and neurodevelopmental disorders (NDDs), such as autism spectrum disorder (ASD) and attention-deficit/hyperactivity disorder (ADHD), as defined in DSM-5, remain unclear. The core pathological features of AN are a persistent restriction of energy intake and/or purging behavior, an intense fear of weight gain, as well as a disturbance in the self-perception of one’s body shape. BN is characterized by binge eating episodes and inappropriate compensatory behaviors (1). BED, which was newly approved for inclusion in the DSM-5, is defined by recurrent episodes of binge eating associated with guilt, disgust, and marked distress but without compensatory behavior (1). Notably, in both the DSM-5 and the ICD-11, further eating and feeding disorders [pica, rumination disorder, and avoidant/restrictive food disorder (ARFID)] were included in the same chapter. Pica and ARFID usually occur in infancy and early childhood. In contrast to AN and BN, they are not associated with concerns about body weight and shape. AN and BN typically manifest during adolescence, and they are strongly tied to maturation processes during later developmental phases (2).

ASD is a pervasive NDD with an onset in early childhood that is characterized by deficits in social communication and interaction combined with restrictive, repetitive patterns of behavior and/or interests. The presence of symptoms in an early developmental period is mandatory for diagnosis. ADHD, another common NDD, also manifests during infancy. The core symptoms of ADHD include attention deficits, hyperactivity, and impulsiveness (1). Many patients show symptoms of both ASD and ADHD; therefore, a dual diagnosis per the DSM-5 is possible (1). ASD and ADHD are the most prominent examples of childhood onset neuropsychiatric disorders (3). Symptomatic overlaps between ADHD and ASD have been reported in terms of hyperactivity, irritability, social impairment, and inattentiveness (4, 5). The diagnosis of ASD is often delayed, and children are sometimes initially misdiagnosed with ADHD due to its symptoms being present in both disorders (6). It is well recognized that ASD and ADHD may be the bases for the later development of secondary psychiatric complications, such as anxiety and depression (7); however, their roles in the development of EDs have not yet been discussed in depth.

To date, several studies have either addressed the prevalence of certain EDs (AN, BN, and BED) in ASD and ADHD samples or *vice versa* or have analyzed the symptomatic overlap between these disorders. Overlapping features between EDs (predominantly AN) and ASD comprise repetitive and restricted behavior, social withdrawal, and difficulties in understanding another’s mental state (8). ADHD shares symptomatic overlap with EDs, especially BN, in terms of impulsive behavior and disturbed reward encoding, which lead to altered motivational control and attentional biases (9).

Some studies have shown associations between ARFID, pica, and ASD (10, 11), but ASD and ADHD in EDs have mainly been investigated separately from each other. However, some studies have examined all three disorders and reported shared traits between them (12, 13).

While the association between ADHD and ASD is not the focus of this review, the relationship between these two diseases and EDs is systematically reviewed.

### Research Question

To answer the research question, we performed a systematic literature review by focusing on prevalence rates of ASD and ADHD in AN, BN, and BED and *vice versa*. In this review, we did not focus on ARFID, pica, and rumination disorder.

Based on studies that addressed either symptomatic, developmental, or genetic differences and commonalities between NDDs and EDs, our specific objective was to clarify the following question: How common are AN, BN, and BED in ASD and ADHD samples and *vice versa*?

## Methods

We conducted a Medline search via Ovid [Ovid MEDLINE® Epub Ahead of Print, In-Process & Other Non-Indexed Citations, Ovid MEDLINE^®^ Daily, and Ovid MEDLINE^®^ (1946 to 07/13/2019)] and searched for epidemiological data on specific EDs (AN, BN, and BED) in ASD and ADHD. The exact search strategy is presented in **Table 1**. The search was carried out on July 13, 2019.

**Table 1:** Search strategy.

anorexia*.mp. OR ANOREXIA NERVOSA/ or ANOREXIA/ OR bulimia*.mp. OR BULIMIA NERVOSA/ or BULIMIA OR eating disorder*.mp. OR “Feeding and Eating Disorders” OR binge eating*.mp. OR Bulimia/		
AND		
autism*.mp. OR Autistic Disorder OR ASD*.mp. OR Autism Spectrum Disorder/ or Autistic Disorder/ OR Asperger*.mp. OR Autism Spectrum Disorder/ or Autistic Disorder/ or ASPERGER SYNDROME/	OR	attention deficit*.mp. OR Attention Deficit Disorder with Hyperactivity OR attention deficit hyperactivity disorder*.mp. OR ADHD*.mp.
AND		
epidemiology*.mp. OR EPIDEMIOLOGY/ OR incidence*.mp. OR INCIDENCE/ OR prevalence*.mp. OR PREVALENCE/ OR comorbidity*.mp. OR COMORBIDITY/ OR frequency*.mp. OR frequency.mp. OR occurence*.mp. OR occurence.mp.		

mp = title, abstract, original title, name of substance word, subject heading word, keyword heading word, protocol supplementary concept word, rare disease supplementary concept word, unique identifier, synonyms.

We included data on the prevalence of AN, BN, and BED in ASD and ADHD and *vice versa*.

The inclusion criteria were defined as follows: The application of valid instruments to confirm the ED/ASD diagnosis was mandatory. EDs and ASD were diagnosed per either the Structured Clinical Interview for DSM Axis I Disorders (SCID-I), ICD-8, ICD-9, ICD-10, DSM-III-R, DSM-IV, or DSM-5. Additionally, we separately investigated studies that had applied the Autism Diagnostic Observation Schedule, (ADOS-2) questionnaire for the ASD diagnostic process (14). The ADOS-2 (14) is a semi-structured assessment for ASD and is the most widely used and best-validated direct observational measure of ASD characteristics (15). Studies that either only focused on certain autistic traits, applied questionnaires other than the ADOS-2 (14) or the Developmental Diagnostic Dimensional Interview—short version (3Di-sv) (16), or did not receive a clinical diagnosis per the criteria mentioned earlier were not considered.

In the analysis of the prevalence rate of ADHD in the ED samples and *vice versa*, only studies in which the ED diagnosis was established per either the SCID-I, ICD-8, ICD-9, ICD-10, DSM-III-R, DSM-IV, DSM-5, or Kiddie Schedule for Affective Disorders and Schizophrenia (17) were included. ADHD had to have been diagnosed per either the SCID-I, ICD-8, ICD-9, ICD-10, DSM-III-R, DSM-IV, DSM-5, DISC-IV (18), or Kiddie Schedule for Affective Disorders and Schizophrenia (17). Additionally, studies that investigated symptoms of childhood ADHD by applying the Wender Utah Rating Scale (19) were considered separately. Studies that focused only on specific ADHD traits or primarily on obesity and not the EDs listed earlier were excluded.

**Figure 1:**
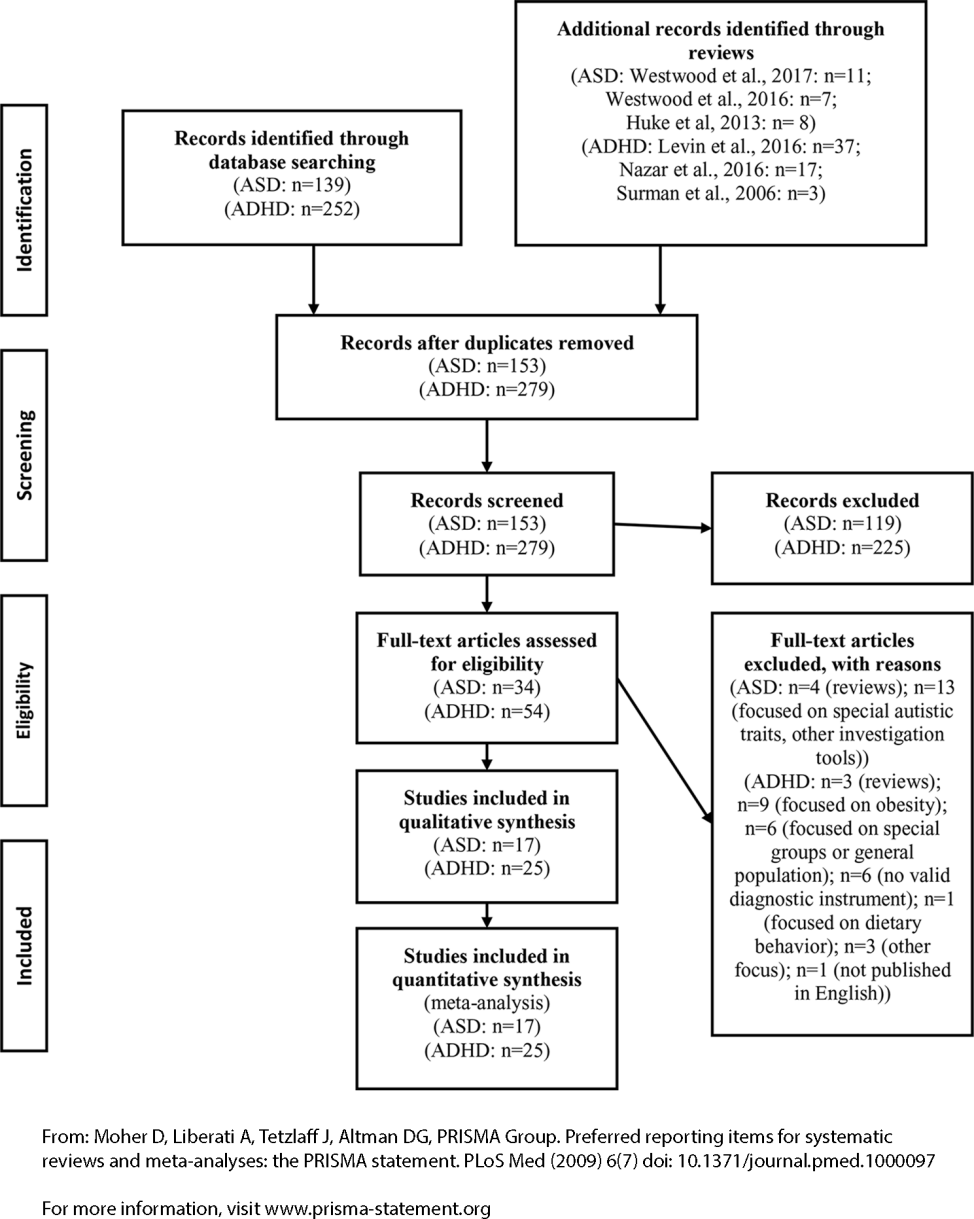
PRISMA flow diagram (selection of studies ASD in EDs, ADHD in EDs, and vice versa).

Other eligibility criteria included being published in a peer-reviewed journal and being available in English. Case reports were not considered. **Figure 1** illustrates the selection of the studies in a PRISMA flow diagram.

## Results

### Autism Spectrum Disorder and Eating Disorders

A total of 139 studies (AN, BN, BED, and ASD) were identified in the Medline search, of which 13 met the inclusion criteria (3, 12, 20–30). We additionally screened the three detected meta-analyses (31–33) carefully for further studies. Huke et al. (31) concentrated on EDs and ASD, which were diagnosed per either the DSM-III-R or DSM-IV or the upcoming DSM-5 or ICD-10 criteria. Westwood et al. focused on ASD traits in EDs per either the Autism Spectrum Quotient (32) or other investigative tools (e.g., the ADOS-2, 3Di-sv, or Ritvo Autism Asperger Diagnostic Scale—Revised) (33). From the reviews, we included four additional studies (34–37), which provided a total of 17 studies for analysis.

**Table 2** lists the results of the systematic literature search for a) ASD in EDs and b) EDs in ASD. The majority of the studies that investigated ASD in EDs focused on AN participants (20, 22–24, 26, 27, 29, 30, 34, 36, 37), while only a few included mixed ED samples (3, 25, 28, 35). The previous studies had mainly examined female participants, with six including only women (3, 26, 29, 30, 34, 35). Some studies primarily focused on adolescents (20, 25, 26, 28, 29, 34, 37), while others included mainly adults (3, 22, 24, 27, 30, 35, 36). Five studies were based on the same Swedish community sample; therefore, to calculate the prevalence rate, we only considered the average prevalence rate of all five studies (16.0%) (20, 22, 24, 36, 37) to avoid biasing effects.

**Table 2:** Previous prevalence studies in patients with EDs (AN, BN, BED) and ASD.

Study	Sample size	Diagnosis	Age in years (mean ± SD)	Diagnostic tools	Results
a) ASD in ED					
1. Westwood et al. (29) cross-sectional	40 (100% females)	AN	15.58 ± 1.35 (n = 19 below cutoff ADOS-2) 14.86 ± 1.62 (n = 21 above cutoff ADOS-2) (12–18 years)	AN: ICD-10, DSM-5, EDE-Q ASD: ADOS-2 Module 4, 3Di-Sv	4 AN cases (10%) received ASD diagnosis; all of the restrictive type 21 AN cases (52.5%) scored above cut-off ADOS-2
2. Westwood et al. (30) cross-sectional	60 (100% females)	AN	26.5 (n = 14 HAS) 23 (n = 24 SCAS) 22 (n = 23 NAS) (18–47 years)	AN: DSM-5, EDE-Q ASD: ADOS-2 Module 4, RAADS-R	14 AN cases (23.3%) scored above cut-off ADOS-2
3. Postorino et al. (26) cross-sectional	30 35 (100% females)	AN HC	14.19 ± 1.56 13.6 ± 1.61 (10–17 years)	AN: DSM-5; EAT-26; EDI-3 ASD: ADOS-2 Module 3 or 4, AQ	3 AN cases (10%) scored above cut-off ADOS-2
4. Bentz et al. (34) cross-sectional	43 28 41 (100% females)	AN AN-RE HC	16.1 ± 1.5 18.4 ± 1.6 17.7 ± 2.2 (14–22 years)	AN: ICD-10, EDE ASD: ADOS-2 Module 4	7 AN cases (16%) scored above cut-off ADOS-2 6 AN-RE cases (21%) scored above cut-off ADOS-2 0 HC (0%) scored above cut-off ADOS-2
5. Koch et al. (23) retrospective cohort study	5006 (93% females)	AN	age at diagnosis <16: 2,336; ≥16: 2,670	AN: ICD-8, ICD-10 ASD: ICD-8, ICD-10	Probands with a first diagnosis of AN with elevated risk of receiving a second diagnosis of ASD (HR=15.08) 225 AN cases (4.5%) received ASD diagnosis
6. Mandy and Tchanturia, (35) cross-sectional	7 2 1 (100% females)	AN EDNOS BN (pre-selected sample)	26.4 ± 6.49 (19–38 years)	ED: DSM-IV ASD: ADOS-2 Module 4	5 ED cases (50%) (pre-selected sample) scored above cut-off ADOS-2
7. Rhind et al. (28) cross-sectional	87 56 1 1 5 (91% females)	AN EDNOS-AN BN Possible ED No ED	16.90 ± 2.13 (13–21 years)	ED: DSM-IV, DAWBA ASD: DSM-IV, DAWBA, SAS, SDQ	6 ED cases (4%) with a possible (n=5) or definite (n=1) ASD diagnosis
8. Anckarsäter et al. (20) restrospective	50 (96% females) 50	AN HC		AN: DSM-III-R, DSM-IV ASD: DSM-IV, ASDI, AQ, TCI, neurocognitive tests	14 (28%) AN cases met DSM-IV criteria for ASD (1 autistic disorder, 4 Asperger’s syndrome, 9 PDDNOS) as compared with 1 HC subject (PDDNOS)
9. Pooni et al. (25) cross-sectional	22 24 20 (86.4%, 87.5% and 20% females)	17 AN, 3 atypical AN, 1 BN, 1 FAED TD ASD	13.0 ± 1.6 13.0 ± 2.4 11.6 ± 2.0 (8–16 years)	ED: DSM-IV-TR, ICD-10 ASD: DSM-IV-TR, ICD-10, 3Di-sv, DAWBA, SDQ, RBS-R	1 ED case (4.5%) received ASD diagnosis ASD diagnosis no more common in ED than in TD Repetitive and stereotyped behavior more often observed in ED compared with TD Trend toward greater autistic social impairment in ED
10. Wentz et al. (3) cross-sectional	30 (100% females)	21 AN 9 BN	27.4 ± 8.4 (18–56 years)	ED: DSM-IV, SCID I, ASD: DSM-IV, ASD-I	7 ED cases (23%) had ASD (all), 5 ED cases (17%) had AD/HD (all BED type)
11. Råstam et al. (27) retrospective	51 51 (94% females)	AN HC	21 24	AN: DSM-III-R, DSM-IV, SCID-I ASD: DSM-III-R and DSM-IV	Study 2: 10 AN cases (20%) met ASD criteria Study 3: Nine AN (18%) subjects and 1 HC (2%) met the criteria for ASD
12. Nilsson et al. (24) retrospective	51 51	AN HC	24	AN: DSM-III-R, DSM-IV ASD: DSM-III-R, DSM-IV, ASDI	9 AN cases (18%) and 1 HC met ASD criteria
13. Nilsson et al. (36) retrospective	51 51 (94% females)	AN HC	21	AN: DSM-III-R, DSM-IV ASD: DSM-IV	15 AN cases (29%) were diagnosed as having “empathy disorder” (defined by Gillberg et al.); 6 of these met DSM-IV criteria for Asperger’s disorder
14. Gillberg et al. (22) retrospective	51 51 (94% females)	AN HC	21	AN: DSM-III-R, SCID ASD: DSM-III-R, criteria for Asperger’s syndrome by Gillberg (1989)	15 AN (29%) had an empathy disorder (as compared with 4% in the HC group) 6 AN (12%) met criteria for Asperger’s syndrome 37% of the AN group had either a cluster C personality disorder or a diagnosis of ASD, as compared with 10% of the HC group
15. Råstam et al. (37) cross-sectional	51 51	AN HC	16	AN: DSM-III-R ASD: DSM-III-R	1 AN (2%) diagnosed with Asperger’s syndrome, 1 girl with PDD, 2 girls had histories suggestive of HFA
b) EDs in ASD					
1. Karjalainen et al. (12) cross-sectional	74 45 109 (44% females)	ASD ASD+ ADHD ADHD	31.75 ± 9.29 (19–60 years)	ED: SCID-I, EAT ASD: SCID-I DSM-IV	18 cases (7.9%) had a current or previous ED [ASD: AN, n = 5 (6.7%); BN: n = 2 (2.7%); BED: n = 1 (1.4%); ADHD: AN: n = 2 (2.8%), BN, n = 0 (0.0%), BED, n = 7 (6.4%); ASD+ADHD: AN, n = 1 (2.2%)]
2. Koch et al. (23) retrospective cohort study	12606 (21% females)	ASD	age at diagnosis: <16: 10,851; ≥16: 1,755	ED: ICD-8, ICD-10 ASD: ICD-8, ICD-10	Probands with a first diagnosis of ASD with elevated risk of having a second diagnosis of AN (HR = 5.3)
3. Bölte et al. (21) cross-sectional	71 32 (28% females)	autism Asperger syndrome	19.7 ± 7.8 (10.1–39.9 years)	ED: clinical diagnosis, DSM-IV ASD: DSM-IV, ADI-R, ADOS	28% of male individuals had a BMI in the 5th percentile or below A clinical diagnosis of AN was not met by any proband

SD, standard deviation; HC, healthy controls; TD, typically developed; ED, eating disorder; AN, anorexia nervosa; BN, bulimia nervosa; BED, binge eating disorder; AN-RE, recovered from anorexia nervosa; EDNOS, eating disorder not otherwise specified; FAED, food avoidance emotional disorder; ASD, autism spectrum disorder; HFA, high-functioning autism; PDDNOS, pervasive developmental disorder not otherwise specified; ADHD, attention-deficit/hyperactivity disorder; BMI, body mass index; ADOS, Autism Diagnostic Observation Schedule (14); SCID, Structured Clinical Interview for DSM; DSM, Diagnostic and Statistical Manual of Mental Disorders; ICD, International Classification of Diseases; EDE, Eating Disorder Examination (38); EDE-Q, Eating Disorder Examination Questionnaire (39); 3Di-sv, Developmental Diagnostic Dimensional Interview-short version (16); RAADS-R, Ritvo Autism Asperger Diagnostic Scale—Revised (40); EAT, Eating Attitudes Test (41); EDI, Eating Disorder Inventory (42); DAWBA, Development and Well-Being Assessment (43); SAS, Social Aptitutes Scale (44); SDQ, Strengths and Difﬁculties Questionnaire (45); ASDI, Asperger Syndrome Diagnostic Interview (46); AQ, Autism Spectrum Quotient (47); TCI, Temperament and Character Inventory (48); RBS-R, Repetitive Behavior Scale—Revised (49); ADI-R,Autism Diagnostic Interview revised (50); HR, hazard ratio.

The reviewed studies showed that, on average, 26.5% [with the exclusion of the preselected sample study (35) 25.4%] of the ED patients scored above the cutoff of the ADOS-2 (26, 29, 30, 34, 35) indicating the presence of important ASD symptoms.

On average, 4.7% of the ED (AN, BN, and BED) patients received an ASD diagnosis per either the DSM-III-R, DSM-IV, DSM-5, or ADOS-2 (14) in combination with the 3Di-sv (16) (3, 20, 22–25, 28, 29, 36, 37). Because most of the reviewed studies focused on AN, we calculated the prevalence rates for this diagnostic category separately. When including only AN participants, the prevalence rate was also 4.7% diagnosed with ASD (20, 22–24, 27, 29, 36, 37).

Literature focusing on EDs in ASD samples is scarce. Karjalainen et al. (12) investigated a sample of 228 adults with an ADHD and/or an ASD diagnosis. For the entire sample, 7.9% had either a current or a previous ED. AN predominantly occurred in ASD (6.7% of ASD patients with an AN diagnosis), while BED was more often diagnosed within the ADHD group (6.4%). A Danish nationwide register-based cohort study showed that participants with a first diagnosis of ASD have an elevated risk of having a second diagnosis of AN (HR = 5.3). However, the risk of comorbid ASD did not differ from the risk of comorbid major depression in AN probands, and it was proposed to be nonspecific (23). Bölte et al. (21) analyzed 103 patients with ASD and found that 28% of male individuals had a body mass index in either the fifth percentile or below. However, the results indicated that the link was inconsistent and partly mediated by hyperactivity (21).

### Attention-Deficit/Hyperactivity Disorder and Eating Disorders

A total of 252 studies (AN, BN, BED, and ADHD) were initially identified, of which 16 met the inclusion criteria (3, 12, 51–64). Additionally, we screened the three detected meta-analyses (9, 65, 66) and included nine further studies (9, 65–75).

**Table 3** presents the results of the systematic literature search for a) ADHD in EDs and b) EDs in ADHD.

**Table 3:** Previous prevalence studies in patients with EDs (AN, BN, BED) and ADHD.

Study	Sample size	Diagnosis	Age in years (mean ± SD)	Diagnostic tool	Results
a) ADHD in EDs					
1. Sala et al. (59) cross-sectional	73 (100% females)	ED (AN-R; AN-BP, BN)	28.07 ± 7.30 (15–50 years)	ADHD: DSM-IV-TR, WURS, BADDS ED: DSM-IV-TR, EAT-40, BITE, EDI-2, BIS-10	13 (18%) with comorbid ADHD (3 AN-R subtype, 9 AN-BP subtype, 1 BN) as to DSM-IV
2. Welch et al. (73) cross-sectional	850 (95.4% females) 8,500	BED HC	22 (14–72 years)	ADHD, ED: ICD-9, ICD-10	14 BED cases (1.7%) had comorbid ADHD, 51 (0.6%) in the HC group
3. Welch et al. (62) cross sectional	101 529 (91% females)	AN EDNOS BN	15.2 ± 1.7 (f) 14.9 ± 1.6 (m)	ADHD, ED: DSM-IV, ICD-10	10 ED cases (1.6%) with a previous ADHD diagnosis; ADHD was present in 4 boys and 6 girls (6.9 vs. 1.0%) in the ED group
4. Seitz et al. (60) cross-sectional	57 40 (100% females)	BN HC	20.8 ± 4.82 21.2 ± 3.99 (15–35 years)	ADHD: DSM-IV, WRI, ADHD-SB, WURS-k, BN: SCID-I, EDI-2, SIAB-EX,	12 BN cases (21%) met clinical cutoff for previous childhood ADHD according to WURS compared with 2.5% of HC 14 BN cases (24.5%) scored above the cutoff of the ADHD-SB compared with 5% of HC 6 BN cases (10.5%) received adult ADHD diagnosis (DSM-IV) according to WURS-k, ADHD-SB and an expert interview
5. Yilmaz et al. (71) cross-sectional	86 (100% females)	BN	24.7 ± 6.7	ADHD: WURS BN: DSM-IV, EDE-12, SCID-I	20 BN cases (23.3%) met clinical cutoff for childhood ADHD according to WURS
6. Yates et al. (63) cross-sectional	55 97 37 (100% females)	AN-R AN-BE BN	<18 years to early adults	ADHD: DSM-IV, SCID-I, MINI ED: DSM-IV, SCID-I, SIAB	10 ED cases (5.3%) met criteria for ADHD diagnosis (1 AN-RE, 9 AN-BE subtype,d or BN)
7. Wentz et al. (3) cross-sectional	30 (100% females)	21 AN 9 BN	27.4 ± 8.4 (18–56 years)	ADHD: DSM-IV, ADHD-RS ED: DSM-IV, SCID-I	5 ED cases (17%) had AD/HD diagnosis; all of the binge eating/purging AN type
b) ED in ADHD					
1. Karjalainen et al. (12) cross-sectional	74 45 109 (44% females)	ASD ASD+ ADHD ADHD	31.75 ± 9.29 (19–60 years)	ADHD: SCID-I DSM-IV ED: SCID-I, EAT	18 cases (7.9%) had a current or previous ED [ASD: AN, n = 5 (6.7%); BN: n = 2 (2.7%); BED: n = 1 (1.4%); ADHD: AN: n = 2 (2.8%), BN, n = 0 (0.0%), BED, n = 7 (6.4%)]
2. Gorlin et al. (56) cross-sectional	204 (50% females) 929	ADHD No ADHD	34.9 ± 13.4 41.1 ± 14.3	ADHD, ED: DSM-IV, SCID-I	19 cases (9.3%) of ADHD patients with comorbid ED 3.8% of patients without ADHD
3. Reinblatt et al. (70) cross-sectional	109 (47% females)	ADHD	10.8 ± 3.7	ADHD: DSM-IV-TR, K-SADS ED: DSM-IV, C-BEDS,	Association between ADHD and BE was statistically significant (OR = 16.1)
4. Bleck et al. (53) Retrospective	575 (51% females)	ADHD	21.8 (18–27 years)	ADHD, ED: DSM-IV	Patients with clinical ADHD more likely to present clinical ED (OR = 2.81) and levels of restrictive (OR = 4.92) and bulimic behaviors (OR = 8.14)
5. Kessler et al. (69) cross-sectional	525 (51% females)	ADHD	13–17 years	ADHD: CIDI, DSM-IV, K-SADS-PL ED: DSM-IV, CIDI	ADHD diagnosis associated with higher lifetime prevalence of ED (OR = 3.2)
6. Edvinsson et al. (55) cross-sectional/ retrospective	168 (46% females)	ADHD	34.4 ± 9.6 (18–57 years)	ADHD, ED: SCID-I, DSM-IV	Lifetime prevalence of ED (both AN and BN) in ADHD women 21.8 and 0% in men
7. Yoshimasu et al. (64) retrospective	343 712 (25% females)	ADHD TD	19 years	ADHD, ED: DSM-IV-TR, based on teacher/parent questionnaires, school records, and medical records	ADHD associated with increased risk of ED diagnosis by age 19 compared with those without ADHD (HR = 5.68)
8. Gau et al. (67) cross-sectional	186 185 (20% females)	ADHD TD	12.9 (11–17 years)	ADHD: DSM-IV, K-SADS-E ED: K-SADS-E	3 (1.6%) of patients with persistent ADHD vs. 0% of TD with an ED (not significant)
9. Biederman et al. (51) 11-year prospective	96 91 (100% females)	ADHD TD	11 ± 3.2 12 ± 2.8 (6–18 years)	ADHD: DSM-III-R, DSM-IV, SCID-I K-SADS-E ED: K-SADS-E	Increased lifetime risk of developing an ED in ADHD group (HR = 3.5) compared with TD, especially BN (HR = 5.2)
10. Mikami et al. (57) 8-year prospective	432 264 (23% females)	ADHD TD	16.4 (7–10 years)	ADHD: DSM-IV, SNAP-IV, DISC-III/IV ED: EDI-II, DISC-IV	No youth met BN criteria; ADHD youth with more BN symptoms (girls > boys)
11. Cumyn et al. (54) cross-sectional	335 112 (40% females)	ADHD TD	(17–74 years)	ADHD: DSM-IV, SCID-I, WURS, CAARS ED: DSM-IV, SCID-I	No relationship between ADHD and EDs
12. Mikami et al. (58) 5-year prospective	127 82 (100% females)	ADHD TD	9.5 (6–12 years)	ADHD: DSM-IV DISC-IV, SNAP ED: EDI-II, EAT, DISC-IV	No girl met diagnostic criteria for BN. Girls with ADHD-C in childhood at risk for BN behaviors in adolescence
13. Ghanizadeh et al. (68) cross-sectional	81 (17% females)	ADHD	8.7 ± 3.07 (5–18 years)	ADHD: K-SADS-PL, DSM-IV ED: K-SADS-PL	No relationship between ADHD and EDs
14. Biederman et al. (52) 5-year prospective	123 112 (100% females)	ADHD TD	11.7 (6–18 years)	ADHD: DSM-III-R, SCID-I, K-SADS-E ED: K-SADS-E	Girls with ADHD with increased risk of developing an ED (HR = 3.6), and BN specifically (HR = 5.6)
15. Sobanski et al. (61) cross-sectional	70 70 (46% females)	ADHD TD	36.8 ± 9.0 39.8 ± 10	ADHD: DSM-IV, SCID-I, WURS-k, BADDS ED: DSM-IV, SCID-I	8 ED cases (11.4%; 5 BE, 3 BN) in ADHD 1 ED case (1.4%) in TD
16. Biederman et al. (74)	219 (37% females) 215	ADHD TD	37.6 ± 10.5 38.7 ± 4.2	ADHD, ED: DSM-III-R (SCID), K-SADS-E	3 AN and 9 BN cases in ADHD group 0 AN and 1 BN case in TD
17. Biederman et al. (72) cross-sectional	280 (50% females) 242	ADHD TD	11.2 ± 3.4 (f) 10.5 ± 3 (m) 12.2 ± 3 (f) 11.6 ± 3.7 (m)	ADHD, ED: DSM-III-R, K-SADS-E	2 BN cases in ADHD group 0 ED cases in TD
18. Biederman et al. (75) cross-sectional	101 (42% females) 207	ADHD TD	39.3 ± 10 (f) 36.9 ± 8.4 (m) 38.0 ± 6.9 (f) 40.1 ± 7.1 (m)	ADHD, ED: DSM-III-R, K-SADS-E	7 BN cases in ADHD group 3 BN cases in TD group

SD, standard deviation; ED, eating disorder; AN, anorexia nervosa; AN-R, anorexia nervosa—restrictive subtype; AN-BP, anorexia nervosa-binge/purge subtype; BN, bulimia nervosa; BED, binge eating disorder; EDNOS, eating disorder not otherwise specified; ADHD, attention-deficit/hyperactivity disorder; TD, typically developed; OSFED, otherwise specified feeding or eating disorder; DSM, Diagnostic and Statistical Manual of Mental Disorders; ICD, International Classification of Diseases; SCID, Structured Clinical Interview for DSM; WURS, Wender Utah Rating Scale (76); BADDS, Brown Attention Deficit Disorder Scale (77); EAT-40, eating attitude test (41); BITE, Bulimic Investigatory Test, Edinburgh (78); EDI, Eating Disorder Inventory (79); BIS, Barrat Impulsivity Scale (80); ASRS, World Health Organization Adult ADHD Self-Report Scale (81); SEDI, Structured eating disorder interview (82); K-SADS, Kiddie Schedule for Affective Disorders and Schizophrenia (17); WRI, Wender–Reimherr Interview (83); ADHD-SB, ADHD self-rating scale (84); EDI-2, Eating Disorder Inventory 2 (85); SIAB-EX, structured interview for anorexic and bulimic disorders for DSM-IV and ICD-10 (86); WURS-k, Wender Utah Rating Scale-Kurzform (19); EDE-12, Eating Disorder Examination (87); MINI, Multi-international Psychiatric Interview (88); SIAB, Structured Interview for Anorexia and Bulimia Nervosa (89); BEDS, Binge Eating Disorder Symptoms Scale (90); DISC-IV, Diagnostic Interview Schedule for Children (18); SNAP, Swanson, Nolan, and Pelham (91); CIDI, World Health Organization Composite International Diagnostic Interview (81); CAARS, Conners’ Adult ADHD Rating Scale (92); BADDS, Brown attention deficit disorder (77); OR, odds ratio; HR, hazard ratio.

The prevalence of ADHD in EDs ranged between 1.6% (62) and 18.0% (59). Comorbid ADHD was more often reported in patients with the AN-binge eating/purging subtype than in the AN restrictive subtype (3, 59, 63). Most of the previous investigations included mixed ED samples (AN, BN, and BED). Only one study included exclusively patients with BED (73), and it found that 1.7% of BED cases had comorbid ADHD. Two studies focused only on participants with BN (60, 71), and they reported that, on average, 22.5% of patients with BN met the clinical cutoff of the Wender Utah Rating Scale questionnaire (76), indicating previous childhood ADHD.

Available studies that have focused on the prevalence of EDs in ADHD are heterogeneous, and their results have varied from no association between EDs and ADHD (54, 58, 68), an association between EDs and ADHD (odds ratio = 16.1) (70), an increased lifetime risk of developing an ED in the ADHD group (51, 52, 64, 69), and a lifetime prevalence of 21.8% of developing an ED in women with ADHD (55).

## Discussion

### Overlap Between Eating Disorders and Autism Spectrum Disorder

A previous systematic review (31) reported increased prevalence rates of ASD in ED populations in comparison with those in healthy controls. The average ASD prevalence of the reviewed studies was 22.9% (31). Importantly, six of the eight considered studies were based on the same Swedish community sample, which could have biased the results and led to the high prevalence rate of ASD in EDs. Huke et al. (31) chose a less stringent definition of the ASD diagnosis, which included patients with “empathy disorder” and histories that were suggestive of high-functioning autism. In our systematic review, we included four additional investigations (23, 25, 28, 29) and detected a lower prevalence rate with, on average, a 4.7% rate of patients with an ED (AN, BN, and BED) or with AN receiving an ASD diagnosis.

The different diagnostic tools (register-based data, clinical diagnosis, various investigation tools), study samples, and methodologies that were used make it difficult to compare the studies and determine the exact prevalence rate of ASD in EDs. Furthermore, diagnostic criteria have changed over the years due to the different versions of the ICD and the DSM. For example, in the DSM-5, autistic disorder, disintegrative disorder in childhood, and Asperger’s syndrome were dimensionally subsumed to ASD. Additionally, the DSM-5 criteria revised AN (e.g., significantly low body weight was newly defined) and BN (e.g., the frequency of binge eating and compensatory behaviors was reduced to weekly), and BED was officially recognized as a formal diagnosis and as the third ED entity (93).

Studies suggest an overrepresentation of ASD in EDs, especially in AN. However, the overlap of EDs with ASD that was found in our study was less than it was with other mental disorders [e.g., 31–89% of patients with AN suffer from depression (94), and 15–69% suffer from obsessive-compulsive disorder (95)]. Furthermore, >50% of ED patients are diagnosed with an anxiety disorder (96). This might properly reflect lower base rates of ASD compared with, for example, depression.

Per the Centers for Disease Control and Prevention, 1 in 59 children (∼1.7%) has been identified with ASD in the general population (https://www.cdc.gov/ncbddd/autism/data.html; retrieval date July 14, 2019). We calculated the prevalence rate as 4.7% of patients with an ED being diagnosed with ASD, which exceeds the prevalence rate for ASD in the general population.

Studies that have investigated the prevalence rates of EDs in ASD samples are scarce and quite heterogeneous; therefore, they are not directly comparable due to the differing investigative tools, age groups, and sample compositions. In particular, because EDs usually occur later in life than ASD and ADHD do, the included children’s studies can only provide information about the ED prevalence rates of their respective age groups.

Associations between EDs and ASD have already been proposed based on clinical observations by Gillberg (97). It has been assumed that autistic impairments might represent a risk factor for both the onset and persistence of EDs (8). One can speculate that social impairments, as they occur with ASD, can lead to reduced social feedback about the disease’s condition as well as special challenges in the therapy, which could result in ED persistence. Stereotypical eating behavior and gustatory, olfactory hypersensitivity may also favor unusual eating habits. Previous research has described overlapping features of EDs and ASD.

An important area of symptom overlap between EDs and ASD concerns impaired neurocognitive functioning. This includes weak central coherence (the ability to see the “big picture” rather than the details), impaired set shifting (the ability to shift from one approach to another fluently and with little difficulty), and difficulties in theory of mind tasks (the ability to attribute mental states) (31, 98, 99). Most studies in EDs have focused on patients with AN. Comparable with individuals with ASD, the weak central coherence in AN is accompanied by a more detail-oriented, so-called “local processing” style and leads to missing the overarching meaning of a situation (8, 100). Weak central coherence is likely a key factor in the shift toward systematized interests in ASD (101). Weak central coherence in AN is most pronounced during the acute state of illness and might also facilitate the shift and restriction of interests but lack the “developmental history” of an onset during early childhood as in ASD. Additionally, executive dysfunction has been reported in EDs, especially in patients with AN and BN (102) as well as in ASD (103). Overall, and because of a lack of longitudinal studies, it is unclear whether neurocognitive deficits are either a risk factor for or a consequence of EDs (98).

Poor theory of mind in AN, however, seems to be limited to the acute state of AN, which could be a consequence of starvation (104). Both disorders show increased social anxiety (105, 106), which is possibly due to difficulties in theory of mind. Social anhedonia (107, 108) and alexithymia (109) have also been described in both, AN and ASD. Again, in AN, what are indeed predisposing characteristics versus what are consequences of the disease have yet to be clarified.

Eating disturbances, such as avoidance of certain food types, sensitivity to food textures, and unusual behaviors at mealtimes, are another overlapping representation in ASD and AN (110). Accordingly, children with ASD are more often hesitant to eat, and their food repertoire is limited (11, 111).

It is difficult to identify the complex relationships between ASD and EDs. It is possible that the overlap of symptoms makes AN and ASD share common underlying cognitive patterns and neuronal pathophysiology. Possible ways in which these traits might be interrelated between ASD and AN are described later. 1) Some of the shared clinical symptoms, such as social anxiety, might be secondary to both. 2) A fearful avoidant and obsessive personality structure (112–114) as well as insecure attachment patterns (115) are associated with an increased risk for AN. These traits are also frequent in ASD patients and might therefore favor the development of AN later. Interestingly, some studies have detected the persistence of cluster C personality disorders after remission from AN (116), meaning that potentially overlapping underlying personality traits may be present between EDs and ASD. However, longitudinal studies are needed to test the hypothesis that shared characteristics of personality functioning increase the risk for ASD as well as AN and form a common ground for both disturbances.

### Overlap Between Eating Disorders and Attention-Deficit/Hyperactivity Disorder

To date, several studies have focused on the relationship between ADHD and EDs. In the reviewed studies, the prevalence rates for ADHD in EDs ranged between 1.6% (62) and 18.0% (59). Comorbid ADHD was more common in the AN-binge eating/purging subtype and BN than in the AN restrictive subtype. In comparison, the prevalence of ADHD in the general population is about 2.5% (117).

Studies that have focused on the prevalence of EDs in ADHD are heterogeneous, and they vary between no relationship between EDs and ADHD and 21.8% of females with ADHD and a lifetime ED history (55).

A previous systematic review found evidence of an association between childhood ADHD and the later development of either an ED or disordered eating. Support for this association was strongest for BN, multiple types of EDs clustered together, and disordered eating (65). Surman et al. (66) performed a systematic analysis on ADHD and BN with data from four case–control studies. Adult women with ADHD had significantly higher rates of BN than those without ADHD (12 vs. 3%, respectively, for one sample and 11 vs. 1%, respectively, for another). Nazar et al. (9) conducted a systematic review and a meta-analysis and found that patients with ADHD have a higher risk of comorbidity with an ED (the pooled odds ratio of diagnosing any ED in ADHD: 3.82), and people with an ED have higher levels of comorbidity with ADHD (the pooled odds ratio of diagnosing ADHD in ED participants: 2.57). The risk of comorbid ADHD in adults with BN was 5.71 (9).

It is difficult to compare previous studies because of the mixed composition of ED samples (AN, BN, and BED), the different age groups, and the various study designs (cross-sectional, retrospective, and prospective). For the analysis of EDs in ADHD samples, it is especially important to compare studies with participants of the same age group to avoid confounding effects when an ED is diagnosed later in development than is ADHD.

The core symptoms of ADHD, inattention, hyperactivity, and impulsivity are often present in EDs (118). Attentional impairment in AN and BN was detected in neuropsychological testing (119). These effects might either result from detrimental effects of malnutrition/starvation or be regarded as a predisposing trait, which increases the risk of developing an ED. Hyperactivity in the form of excessive exercising is common in AN and BN and is associated with compulsivity, perfectionism, and difficulties in affect regulation (120, 121). However, exercising is clearly related to attempting to influence weight and shape. The core features of BN are binge eating and purging behaviors, which can be described as impulsive (122). Previous investigations have shown increased impulsiveness and an impairment of inhibitory control to disease salient stimuli in patients with BN (123). In addition, studies have indicated that impulsivity in childhood, as opposed to inattentiveness and hyperactivity, is most predictive of adolescent BN pathology (57, 58). Apart from core ADHD symptoms, EDs and ADHD also share related symptoms, such as depression, anxiety, and low self-esteem (124, 125). Although there seems to be some overlap in clinical symptoms between EDs and ADHD, the relationship between these disorders needs further investigation. Longitudinal studies are needed to determine which factors precede the diseases (EDs and ADHD) and which should be regarded as their consequences.

### Overlap Due to Shared Comorbidities?

When analyzing shared traits between EDs, ASD, and ADHD, it should be considered that additional shared common comorbidities might result in clinical and symptomatic overlap. Comorbidities are the rule rather the exception in patients with EDs (126), ASD (127, 128), and ADHD (129) alike. For example, anxiety disorders and mood disorders are frequently reported comorbidities of EDs (130, 131), ASD (132), and ADHD (129). In addition, impulse control problems were reported in patients with EDs (especially BN and BED) and patients with ADHD. “Multi-impulsive behaviors,” including alcohol abuse (133) and drug abuse (134), have been observed in a subgroup of ED patients (135) as well as in ADHD (129).

The comorbidity of EDs (37) and ASD (136) with obsessive disorders is well recognized. An obsessive behavior may explain the symptomatic overlap in rigidity and perfectionism (8).

Because of the number of shared comorbidities, it might be difficult to completely determine their influence on symptom overlap between EDs, ASD, and ADHD and the prevalence rates of comorbidity. For future studies, it is therefore essential to carefully measure the rate of major overlapping comorbidities between EDs, ASD, and ADHD.

In addition, it is important to recognize that several symptoms in AN may be a consequence of malnutrition (137). Starvation may also lead to symptoms, such as irritability, depressed mood, decrease in self-initiated activity, and social introversion (138), which might also occur in ASD patients.

### Eating Disorders as a Neurodevelopmental Disorder?

Per the DSM-5, NDDs are characterized by a manifestation of a set of stable perceptive, cognitive, emotional, and behavioral features early in development. NDDs frequently co-occur (1). A required criterion for an NDD is its manifestation early in development. Some previous studies have suggested that patients with EDs, especially those with AN, retrospectively report features that resemble features in NDDs, such as social difficulties in childhood (139). Social problems at age 8 were strongly predictive of EDs’ onset at age 14 (140). Additionally, two studies found an association between childhood impulsivity and the development of BN (57, 58). This may indicate that there are already predisposing traits earlier in childhood for the later development of an ED. However, it is less clear how specific these factors are for EDs. Furthermore, social problems in AN can be attributed to premorbid factors, such as insecure attachment patterns and harm avoidance (115, 126). By contrast, in ASD, they are due to impairment in communication, including the understanding of the conventions of social interaction.

Regarding the overrepresentation of ASD and ADHD in EDs, it could be discussed whether ASD and ADHD themselves might be predisposing factors for the development of EDs, which mainly occur during adolescence (2). Developmental changes, such as puberty, stressful events, and challenges, could trigger ED behaviors (141). Puberty could be considered an especially stressful phase of life for patients with ASD and ADHD. The additional stress of puberty for patients with ASD and ADHD (142, 143) may increase their vulnerability for the development of an ED. In addition, patients with NDDs have an elevated risk for other mental disorders beyond the ED spectrum (129, 132).

Therefore, it appears that NDDs may constitute a vulnerability factor for EDs. As such, EDs might be regarded one of many possible progressive (comorbid) “sequelae” of NDDs. An NDD is not a prerequisite for EDs, and EDs can also occur later in life; therefore, in our view, EDs, in general, should not be considered NDDs, but they could be regarded as a possible later aggravator of an NDD when symptoms evolve from the disease. EDs with a combined NDD may be considered special subgroups of EDs.

## Conclusion

The literature points to considerable comorbidity rates and a symptomatic overlap between certain EDs, ASD, and ADHD. Nevertheless, the question of how much of the overlap is due to shared traits of the disorders per se and how much is due to either shared comorbid conditions or resembling traits has not been fully determined. There is a need for longitudinal studies to answer this question. Clinically, it is important to carefully conduct diagnostic procedures while considering the comorbidity rates and the overlap of symptoms. A thorough diagnostic workup should be conducted to develop individualized treatment strategies. In our view, EDs in general should not be regarded as NDDs. However, in a relevant subgroup of patients, they might be understood as a second-decade sequel of possibly subsyndromal NDDs, when EDs evolve from symptoms and/or psychodynamic problems that are linked to NDDs. EDs with combined NDDs may be considered special subgroups of EDs. To date, studies that have focused on the overlap between all three disorders are scarce. In the future, the risk of developing an ED in individuals who have been diagnosed with either ASD or ADHD should be systematically investigated, preferably in prospective studies. Case studies might add useful information to help derive hypotheses about factors that promote the development of a comorbid ED in adolescence. Prospective imaging studies in individuals at high risk for EDs (both with and without NDDs) could elicit more information about underlying common neurobiological structures. In addition, genetic studies could provide more information about possible overlapping genetic risk factors.

## Author Contributions

KN, SM, AZ, and LT wrote the manuscript. KN, VM, and DE performed literature search. KN, SM, AZ, LT, AJ, VM and DE were crucial involved in the theoretical discussion and the preparation of the manuscript. All authors read and approved the final version of the manuscript.

## Funding

The article processing charge was funded by the German Research Foundation (DFG) and the University of Freiburg in the funding program Open Access Publishing.

## Conflict of Interest

LT: advisory boards, lectures, or travel grants within the last 4 years: Eli Lilly, Janssen-Cilag, Novartis, Shire, UCB, GSK, Servier, Janssen, and Cyberonics. DE was supported by the Berta-Ottenstein-Programme for Advanced Clinician Scientists, Faculty of Medicine, University of Freiburg.

The remaining authors declare that the research was conducted in the absence of any commercial or financial relationships that could be construed as a potential conflict of interest.
